# Intussusception in children under five years of age in Enugu, Nigeria

**DOI:** 10.11604/pamj.supp.2021.39.1.20811

**Published:** 2021-08-06

**Authors:** Beckie Nnenna Tagbo, Uchechukwu Obiora Ezomike, Oluwatoyin Arinola Odetunde, Benedict Onyeka Edelu, Bismarck Christopher Eke, Ogechukwu Francesca Amadi, Ifeyinwa Bernadette Okeke, Okechukwu Ani, Chinedu Michael Chukwubuike, Jason Mathiu Mwenda, Sebastian Okwuchukwu Ekenze

**Affiliations:** 1 Institute of Child Health & Department of Paediatrics, University of Nigeria Teaching Hospital, Enugu, Nigeria,; 2 Department of Paediatric Surgery, University of Nigeria Teaching Hospital, Enugu, Nigeria,; 3 Department of Paediatric Surgery, Enugu State University Teaching Hospital, Enugu, Nigeria,; 4 Department of Paediatrics, University of Nigeria Teaching Hospital, Enugu, Nigeria,; 5 Department of Paediatrics, Enugu State University Teaching Hospital, Enugu, Nigeria,; 6 Department of Microbiology, University of Nigeria Teaching Hospital, Enugu, Nigeria,; 7 World Health Organization, African Regional Office, Brazzaville, DRC

**Keywords:** Intussusception, children, rotavirus vaccine, hydrostatic reduction, Nigeria

## Abstract

**Introduction:**

intussusception is the invagination of a segment of the bowel into a distal segment. It occurs predominantly in infants worldwide. Following documentation of increased incidence after introduction of the first rotavirus vaccine (Rotashield, Wyeth-Lederle), it has become a standard recommendation to maintain surveillance for intussusception as newer rotavirus vaccines are introduced into EPI. Nigeria plans to introduce rotavirus vaccine in 2020. Pre-vaccine introduction surveillance will serve as a baseline to understand the epidemiology of intussusception in Nigeria.

**Methods:**

from 2013 to 2017, prospective enrolment of under five children with intussusception was done following the WHO protocol and using the WHO case report form. Only children who met the Pan American Health Organization/World Health Organization (PAHO/WHO) protocol case definition for intussusception were enrolled. These children were monitored until discharge or death. Clinical features and outcome were recorded in the case report form.

**Results:**

a total of 63 cases were enrolled, with age range of 3 to 42 months (median: 6 months, IQR: 5-9 months). Majority were within 4-6 months and 96% were < 12 months old. There were 41 males and 22 females (male to female ratio of 1.9:1). Duration of symptoms before presentation ranged from 2 hours to 15 days (median: 72 hours). Fifty-seven patients had abdominal ultrasound and 52 patients (83%) had surgery. Case fatality rate was 9% and duration of hospitalization ranged from 1 to 30 days (median 10 days, IQR 8-15 days).

**Conclusion:**

intussusception occurred most commonly in infants but well beyond the proposed age for rotavirus vaccination in the population studied. Late presentation and surgical intervention were common. This data provides a good baseline description of the epidemiology of intussusception.

## Introduction

Intussusception is the invagination of a segment of the bowel into a distal segment [1-3]. It can result in obstruction, vascular compromise, and necrosis of the intestine, and may lead to death if untreated. Globally approximately two-thirds of cases occur in children less than 1 year, with a peak incidence from 5 to 9 months of age [4]. Diagnosis is made by air or liquid contrast enema, abdominal ultrasound, or during surgery or autopsy. Some cases resolve spontaneously, but most require hospitalization and ultrasound-guided hydrostatic or surgical reduction.

In 1999, a first generation oral rotavirus vaccine, Rotashield (Wyeth-Lederle), was withdrawn from the US market because of a significantly increased risk of intussusception occurring after its administration [5]. A population-attributable risk of 1 case of intussusception per 10,000 vaccine recipients (range, 1 case per 5,000-12,000) was estimated; the highest risk (> 30-fold) was observed 3-7 days after the first dose of the vaccine [6, 7]. The unexpected association between Rotashield and intussusception, which led to the withdrawal of the vaccine, delayed public health efforts to reduce the burden of rotavirus worldwide. Before introduction of the currently available rotavirus vaccines, large pre-licensure trials were required to demonstrate their safety. Based on the heightened awareness regarding intussusception, pre-clinical safety trials for the current World Health Organization (WHO) pre-qualified rotavirus vaccines, Rotarix (GlaxoSmithKline) and RotaTeq (Merck & Co.), were assessed for intussusception. For RotaTeq, a 42 day post-dose 1 window was assessed, and for Rotarix, a 30 day post-dose 1 window was assessed. Each study was done among > 60,000 infants and identified no vaccine attributable intussusception risk [8, 9].

After RotaTeq was introduced in the US in 2006, a post-licensure safety study did not show an increased risk of intussusception [10]. Recent studies from the US, Australia, Brazil, and Mexico, however, have shown 1- to 5-fold increased risk of intussusception following vaccination with Rotarix and RotaTeq [11-14]. This risk is 5-10 times lower than the risk seen with Rotashield. No association between Rotarix vaccine and intussusception was observed in a recent evaluation in 7 countries in sub-Saharan Africa [15]. The physiological reason behind the association of rotavirus vaccines and intussusception is not well understood. In most countries, reliable estimates of the incidence of intussusception are not available, and differing case definitions makes comparisons among studies difficult. Sufficient data on intussusception-associated hospitalizations in Nigeria are currently scarce. Therefore, we aim to describe the epidemiology of intussusception-associated hospitalizations among children in Enugu, Southeast Nigeria. Because the public health impact of rotavirus vaccination could be substantial in settings like Nigeria, where diarrhoea-related morbidity and mortality is high, assessment of the baseline epidemiology of intussusception will be useful to public health officials and to the Federal government of Nigeria in making decisions about vaccine introduction.

The primary objective of the current study was to describe the pre-vaccine introduction epidemiology (e.g., age distribution and seasonal patterns) of intussusception hospitalizations among children < 5 year of age in Enugu, Nigeria. The secondary objectives were to determine the proportion of intussusception-associated hospitalizations that required surgical treatment and the proportion that results in death.

## Methods

This is a prospective, active surveillance of infants with intussusception presenting to the sentinel hospital from January 2013 to December 2017. The hospitals are University of Nigeria Teaching Hospital (UNTH) and Enugu State Teaching Hospital (ESUTH), both in Enugu State (However the later withdrew from the study in 2016). Inclusion criteria for enrolment were children who were < 5 years of age and who met the PAHO/WHO protocol case definition for intussusception [16]. Surveillance staff, working with paediatricians and paediatric surgeons identified intussusception cases that met the inclusion criteria, either directly at presentation or through review of registers in the children´s emergency and Paediatric surgical wards. A case report form was then completed for each eligible case identified and a clinical register was completed. Data collected included demography, main symptoms and findings on examinations; investigations, treatment and outcomes. Data was then transferred to a database for storage and subsequent analysis using the MS excel and GraphPad Prism software version 7.05. Intussusception trends and proportions were generated and data presented using descriptive statistics in tables and charts.

## Results

**Figure 1:**
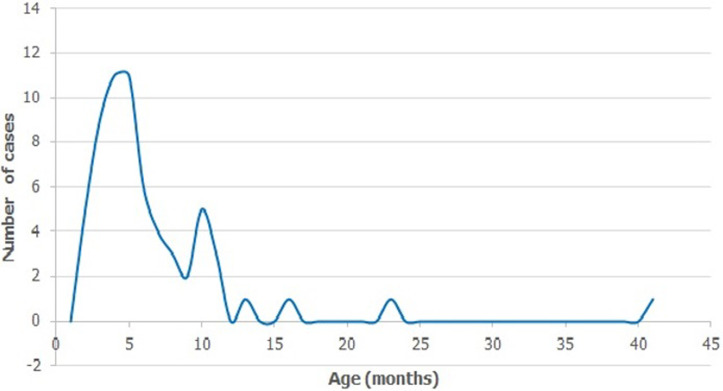
age distribution of intussusception cases 2013-2017

**Figure 2:**
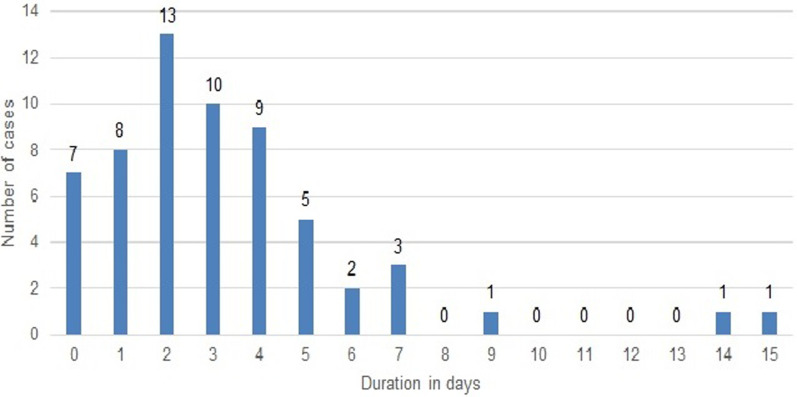
symptom duration before presentation (days)

A total of 63 cases that met the inclusion criteria were enrolled from 2 hospitals from 2013 to 2017. Tewlve cases presented at ESUTH while 51 cases presented at UNTH. The age ranged from 3 to 42 months with a median age of 6months and interquartile range of 5-9 months (Figure 1). Majority of cases occurred in the 4-6 months (49%) and 7-12 months (37%) age strata giving a total of 86% of cases occurring at 4 to 12 months (Table 1). Only 8% were < 3months old and 6% were > 12 months of age. There were 41 males and 22 females with a male to female ratio of 1.9:1. The yearly distribution showed 13, 18, 5, 13 and 11 cases in 2013, 2014, 2015, 2016 and 2017 respectively (3 missing). Duration of symptoms before presentation ranged from 1 to 15 days with a median of 3 days (IQR 1-4 days), (Figure 2). Fifteen (25%, n=60) patients had symptoms < 24 hours before presentation.

**Table 1:** clinical and lab variables among the study population

**Age**	**N=63**	**%**	**Duration from presentation to surgery/intervention**	**N=61**	**%**
< 3 months	5	8	<24 hours	16	26
4-6 months	31	49	>24-<48 hours	13	21
7-12 months	23	37	>48-<72 hours	10	16
>12 months	4	6	>72 hours	22	36
Total	63	100	Total	61	100
Laboratory investigations*	**N=63**	%	**Discharge Outcome**	**N=58**	%
Plain abdominal x-ray	21	26	Discharged	51	88
Contrast x-ray	3	4	Died	5	9
Ultrasound Scan	57	70	Absconded	1	2
None	1	1	Total	57	100
			Missing	6	
**Mode of management^**	**N=60**	%	**Duration of hospitalization**	**N=60**	%
Bowel resection	31	49	0-7 days	30	51
Hydrostatic reduction	7	11	>7-<14 days	19	32
Reduced at surgery	18	29	>14 days	10	17
Other surgeries	4	6	Total	59	100
Missing	3	5			

* some patients had more than one lab investigation. ^some patients had more than one mode of management

**Figure 3:**
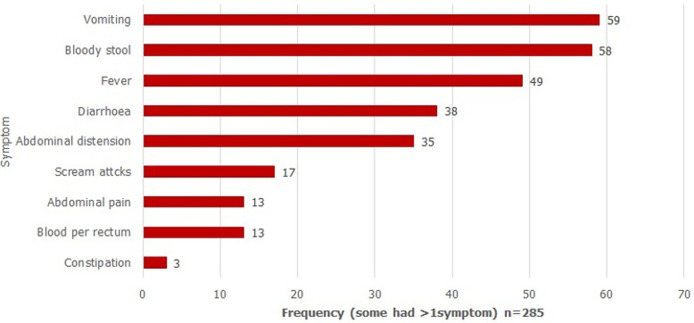
symptom frequency among 64 intussusception cases, 2013-2017 (each patient had > 1 symptom) n = 285

**Figure 4:**
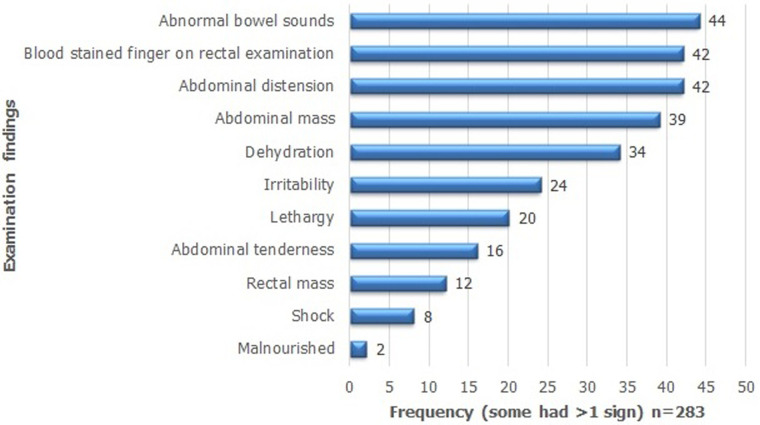
frequency of examination findings among 64 intussusception cases, 2013-2017 (each patient had >1 sign) n = 283

The commonest presenting symptoms were vomiting (21%; 59/285), bloody stool (20%; 58/285), fever (17%; 49/285), diarrhoea (13%; 38/285) and abdominal distension (12%; 35/285); (Figure 3). There were more than one symptom per patient. Among patients with vomiting, majority had 3 to 6 episodes per day, with a range of 1-10 and a median of 4 episodes (IQR 3-6 episodes). Among patients with diarrhoea, episodes per day were mostly 3-5 with a median of 4 episodes (IQR of 3-5 episodes). The commonest findings on physical examination were abnormal bowel sounds (16%; 44/283), blood stained finger on rectal examination (15%; 42/283), abdominal distension (15%; 42/283) and abdominal mass (14%; 39/283) There were more than one sign per patient (Figure 4). Of those with known outcome, 57 patients (89%) had abdominal ultrasound and investigation confirmed intussusception in all cases. In all 63 patients (100%) laboratory investigation showed evidence of intussusception. In 53 cases (83%), surgery confirmed intussusception, comprising bowel resection (31, 48%), open reduction at surgery (18, 28%) and other forms of surgery. Majority had right hemi-colectomy. Seven patients (11%) had successful hydrostatic reduction.

Interval between presentation and surgery/intervention ranged from 1 to 90 hours with a median of 24 hours and IQR of 18-36 hours. Fifty-one (88%) were discharged alive, while 5 died giving a case fatality rate of 9%. Duration of hospitalization was between 1 and 30 days (median 10 days, IQR 8-15 days) 83% of the patients were discharged within 14 days of admission (Table 1). Although Rotarix was available in the private market, only one patient received Rotarix at about 6 months prior to presentation (oral information not card verified).

## Discussion

Although the age of children involved in this study ranged from 3 months to 42 months, majority were aged 4months to 12 months constituting 86% of the cases. This agrees with findings in a previous study in the same population [17] and most other populations where majority of intussusception cases occured in infants [18-21]. In the population studied, the peak age of occurrence was 4-6months which is similar to findings elsewhere [21-25]. This peak occurred later than the proposed scheduled time for rotavirus immunization in the country (6 and 10 weeks). Reports of intussusception at early age in African children is rare [26]. This is worthy of note in the interpretation of post-vaccine introduction data and in comparing it to other countries where rotavirus vaccines are given at a later age. Comparison of intussusception pre- and post-vaccine introduction data should not only be in terms of number of cases but also pattern of occurrence and interval from vaccination time [27]. The males were nearly double the number of females (1.9:1) and this has been a nearly constant observation worldwide [17, 28-30]. The reasons are not quite clearly understood [30]. The yearly distribution did not show any particular trend probably due to frequent industrial strikes by various categories of health workers which in turn could be attributed to political instability and weak governance. There was no seasonal pattern observed too [21].

The observed median duration of symptoms prior to presentation (72 hours) was an improvement compared to a median duration of 4.1 days in a previous study [17] in the same population. It is however, longer than the finding of 1.5days by Moore *et al*. [31] in Brazil and 1 day by Fernandes *et al*. [32] in South Africa. However, Moore´s study included children up to 14 years. The observation of 25% of patients with symptom duration of 24 hours is much less than that observed in a Swiss study [33] where 52% presented in less than 24 hours of symptom onset. Although the health seeking behaviour and awareness is generally sub-optimal in the country of study [34, 35], it is relatively better in the population studied when compared to other sections of the country and rural populations [35]. The location of a Federal Teaching Hospital in the area probably influenced the people´s awareness and health seeking behavior positively. When children become ill, it is common practice for mothers/caregivers to first attempt self-medication, then go to patent medicine dealers, then go to primary health facilities before referral to secondary and tertiary health facilities. Some cases of intussusception upon getting to the State Teaching Hospital were still referred to the Federal Teaching Hospital. This results in a long duration of symptoms before presentation at the hospital of final care and surgery. Such late presentation also affects outcome. The longest duration of symptom before presentation was 15 days.

Most of the patients had abdominal ultrasound scan done (89%). This shows high quality of diagnosis which was based on PAHO/WHO protocol case definition for intussusception [16]. The availability, accessibility and affordability of basic investigation technology is key to prompt diagnosis and good management outcomes for intussusception. But these technologies are not available to many children in rural populations and even when available, they are sometimes not affordable due to high level of poverty in developing regions of the world [40]. Most patients had surgery and it was mostly bowel resection while a few had hydrostatic reduction. This underscores the indispensability of a paediatric surgeon and also highlights the magnitude of late presentation resulting in bowel resection. Morbidity is therefore high and ought to be reduced by early presentation and prompt management [41].

The median interval between presentation and surgery/intervention of 24 hours indicates fairly good management response by the managing staff. Most patients were discharged alive with a case fatality rate of 9% which is not too high in view of relatively late presentation. Case fatality rates of 10 to 33.7% [26] have been reported in African countries including CFR of 23%, 28%, and 33.7% in Nigeria [42], Rwanda [43] and Zambia [44] respectively. However this is higher than what is reported in developed countries where the CFR is usually less than 1-4% [26], where cases present early leading to early intervention. High CFR is usually attributed to delay in patient presentation to hospital and delay in intervention. This results in most patients requiring surgical intervention. It also prolongs hospital stay for the surviving patients. In the current study, the median duration of hospitalization of 10 days is comparable to studies elsewhere [32] but longer than that reported by Buettcher *et al*. [33]. This could still be improved upon by creating awareness among caregivers to seek help early as well as health workers to develop high index of suspicion, early diagnosis and early referral/treatment.

## Conclusion

Intussusception occurs most commonly in infants but well beyond the proposed age for rotavirus vaccination in the population studied. Most patients presented late resulting in surgical intervention in most cases and a moderate CFR. This pre-vaccine introduction data provides a baseline for assessing intussusception occurrence post vaccine introduction. There is need to create awareness among the public and health workers to enhance early presentation to tertiary hospitals for prompt management of cases.

### What is known about this topic

- Global background prevalence of intussusception in children;
- Presentation patterns mostly in developed countries;
- Treatment patterns and age trends in developing countries.

### What this study adds

- Prospective study on prevalence in Enugu;
- Pre-vaccine introduction data on the epidemiology of intussusception in Nigeria;
- Different presentation and treatment pattern/trends in a developing country (Enugu-Nigeria) compared to developed countries.
